# Preparations and Thermal Properties of PDMS-AlN-Al_2_O_3_ Composites through the Incorporation of Poly(Catechol-Amine)-Modified Boron Nitride Nanotubes

**DOI:** 10.3390/nano14100847

**Published:** 2024-05-13

**Authors:** Arni Gesselle Pornea, Duy Khoe Dinh, Zahid Hanif, Numan Yanar, Ki-In Choi, Min Seok Kwak, Jaewoo Kim

**Affiliations:** 1 R&D Center, Naieel Technology, 6-2 Yuseongdaero 1205, 2nd FL, Daejeon 34104, Republic of Korea; porneaarni@naieel.com (A.G.P.); khoedd@gmail.com (D.K.D.); zahidhanif@naieel.com (Z.H.); numanyanar@gm.gist.ac.kr (N.Y.); choiki@naieel.com (K.-I.C.); 2 CMT Co., Ltd., 322 Teheran-ro, Hanshin Intervalley 24 Esat Bldg., Gangnam-gu, Seoul 06211, Republic of Korea; mskwak@cmtcorp.co.kr

**Keywords:** thermal interface materials, thermal conductivity, boron nitride nanotubes, composites, polydimethylsiloxane, fire retardant

## Abstract

As one of the emerging nanomaterials, boron nitride nanotubes (BNNTs) provide promising opportunities for diverse applications due to their unique properties, such as high thermal conductivity, immense inertness, and high-temperature durability, while the instability of BNNTs due to their high surface induces agglomerates susceptible to the loss of their advantages. Therefore, the proper functionalization of BNNTs is crucial to highlight their fundamental characteristics. Herein, a simplistic low-cost approach of BNNT surface modification through catechol-polyamine (CAPA) interfacial polymerization is postulated to improve its dispersibility on the polymeric matrix. The modified BNNT was assimilated as a filler additive with AlN/Al_2_O_3_ filling materials in a PDMS polymeric matrix to prepare a thermal interface material (TIM). The resulting composite exhibits a heightened isotropic thermal conductivity of 8.10 W/mK, which is a ~47.27% increase compared to pristine composite 5.50 W/mK, and this can be ascribed to the improved BNNT dispersion forming interconnected phonon pathways and the thermal interface resistance reduction due to its augmented compatibility with the polymeric matrix. Moreover, the fabricated composite manifests a fire resistance improvement of ~10% in LOI relative to the neat composite sample, which can be correlated to the thermal stability shift in the TGA and DTA data. An enhancement in thermal permanence is stipulated due to a melting point (Tm) shift of ∼38.5 °C upon the integration of BNNT-CAPA. This improvement can be associated with the good distribution and adhesion of BNNT-CAPA in the polymeric matrix, integrated with its inherent thermal stability, good charring capability, and free radical scavenging effect due to the presence of CAPA on its surface. This study offers new insights into BNNT utilization and its corresponding incorporation into the polymeric matrix, which provides a prospective direction in the preparation of multifunctional materials for electric devices.

## 1. Introduction

As our reliance on ever-growing technology deepens, current electronic devices provide better performance, integrability, and convenience. Conversely, the advances in these devices result in the compaction of the integrated circuit (IC) assembly, causing heightened heat flux generation during operation, which needs to be driven out of the system to ensure the highest performance. Analogously, the sharp elevation in heat accumulation increases the likelihood of fire. Thermal interface materials (TIMs) have been utilized to fill the gap between the heat source and heat sink, thereby enunciating the thermal flow to evade the occurrence of heat buildup [1]. Owing to their low cost, lightweight, and processability, polymeric materials have gained much attention in electronic thermal management applications [2]. Conversely, most polymeric materials possess an inherently low thermal conductivity and immense flammability [3,4]. Therefore, it is imperative to develop TIMs with effective thermal conduction and high flame retardancy [5].

One of the widely implemented strategies for developing thermal materials with elevated thermal conductivity and flame retardancy is the incorporation of thermally conductive fillers (e.g., aluminum oxide, aluminum nitride, silver microparticles, etc.) in the polymeric matrix to achieve higher thermal conductivity values [6,7]. A combination of these varieties of fillers was also explored, addressing the individualistic limitations [8]. One of the most commonly employed combinations is AlN and Al_2_O_3_; AlN is commonly used as the larger particle due to its particle shape and size limitation, while Al_2_O_3_ is utilized for the smaller particles, obtaining an effective packing condition and attaining the optimal percolation threshold. Hence, upon approaching the percolation threshold concentration, there is not much that can be done to increase the thermal conductivity [9]. One of the discovered strategies to augment the thermal conductivity without further increasing the filler concentration is the assimilation of high-aspect-ratio fillers (e.g., carbon nanotubes, silver nanowires, graphene, etc.) with intrinsically high thermal conductivity in relatively small quantities, as they can effectively establish thermal pathways when positioned in between the crevices of the bigger particles [10,11,12]. Recently, Boron nitride nanotubes (BNNTs), a CNT derivative, have gained popularity, not only due to the traditional attributes that it shares with CNT, such as geometrical, thermal conductivity, and mechanical properties, but also the demonstration of exceptional assets such as electrical insulation and oxidative resistance [13,14,15]. These probed BNNTs are favored as a nanofiller candidate for the preparation of thermal interface materials, constituting contributions on the thermal conductivity and fire repellence performance. However, due to strong Van der Wall interactions, BNNT nanofillers are susceptible to aggregation [16]. Thus far, BNNT utilization on TIMs is still in its infancy due to its surface chemical inertness that weakens its interaction with solvents and polymeric media, which results in its poor dispersive behavior, leading to severe BNNT agglomeration in the polymeric composite matrix [17]. The BNNT clustering not only influences the abatement of the thermal pathway formation but also degrades the diffusion boundary layer of the polymer’s volatile compounds during combustion. Therefore, surface modification and an intricate fabrication strategy must be implemented to attain good dispersion and compatibility with the polymeric matrix that will consequently emulate improvements in the thermal conductive performance of the composite material [18]. Moreover, due to the limited functional groups on the BNNT interface, surface treatment is still a challenge. There have been some reports of surface treatment of BNNTs to improve the dispersibility and facilitate polymer matrix compatibility. However, these strategies typically involve expensive materials and intricate processes. Thus, simultaneous fulfillment of dispersibility, functionality, and processability is still a formidable hurdle. 

Recently, polydopamine (PDA) has gained attention as a surface-treating agent due to its excellent adhesive properties; hence, its high price impedes its industrial application [19]. Alternately, it was recognized that catechol and amine groups were the key factors in the polymeric oxidation of dopamine to form strong adhesion [20]. Thus, catechol (CA) and polyamine (PA) polymerization exhibits adhesive properties, similar to those of PDA [21]. Herein, we present a simple and practical strategy for surface treating BNNTs with CA and PA to instigate effective dispersion and polymeric matrix interfacial compatibility, compounding both superior thermal conductivity and flame retardancy improvements [21]. The surface-treated BNNT was introduced into the PDMS/AlN/Al_2_O_3_ composite to enhance the thermal conductivity while simultaneously reducing fire hazards. The effective BNNT dispersion leads to the formation of bridges between the other fillers, thereby constructing a thermally conductive network in the polymeric matrix. In addition, the surface modifications in thermally conductive nanofillers not only contribute to their improved dispersion and polymeric compatibility, which translate into a thermal conductivity enhancement, but also augmented the flame retardation of the resulting composite. BNNTs serve as a bridge to enhance phonon transport by connecting microparticles, thereby improving the overall thermal conductivity and reducing the bulk thermal resistance. The flame retention improvement can be associated with the synergy between the inherent thermal stability of BNNTs, char formation from the combusted polymer, and free radical scavenging effects. The incorporated nanofiller forms a barrier network, thereby inhibiting the heat and oxygen penetration and exposure of volatile products. The CAPA-enriched BNNT establishes an apparent flame-retardant enhancement, owing to the free radical scavenging effect, suppressing the occurrence of further ignition and combustive reactions, thereby restraining the spread of fire. Consequently, this concept provides new information on the BNNT utilization and structurization to produce a multifunctional polymeric composite material. To the best of my knowledge, this is the first attempt to showcase multifunctional materials with the aid of BNNTs, fostering contributions in thermal conductivity and fire-resistive performance of thermal interface materials.

## 2. Materials and Methods

### 2.1. Materials

AlN particles were acquired from Thrutek. Al_2_O_3_ microparticles were purchased from Denka Korea. Catechol (CA), tetraethylenepentamine (PA), and tannic acid (TA) were obtained from Deajung Korea. Tris HCl (1M) was bought from Hanlab. The BNNT powder (diameter: 30–50 nm, length: ~10 µm, Purity: >80%) was synthesized through a thermochemical reaction of boron powder with nitrogenous gases. The purity of BNNT suggests the fractional concentration of nanotubes by weight, while the remainder is h-BN nanoparticles and nanoflakes. PDMS resin, hardener, retardant, and curing agent were supplied by MQ Korea.

### 2.2. Preparation of BNNT-CAPA

First, 1 g of BNNT was added to 1 L of tannic acid (TA) aqueous solution and was subjected to tip sonication for 10 min. This process disperses BNNTs to eliminate agglomeration and ensure even surface coating. Then, 3 g of CA was added to the solution followed by 1 g of PA, and the solution was then tip sonicated for 1 h at 80 °C. Afterward, the BNNT changed its color to a pale-orange color; the functionalized BNNT was then collected through centrifugation and washed accordingly.

### 2.3. Preparation of PDMS/AlN/Al_2_O_3_/BNNT-CAPA Composites

The thermal composite was assembled by simply mixing with the different fillers through sequential vortex mixing with a paste mixer. Firstly, a certain amount of BNNT-CAPA was initially blended with IPA and PDMS resin to ensure effective dispersion; the solvent was then evaporated before the incorporation of the other fillers. The remaining filler (Al_2_O_3_ and AlN) was then incorporated into the BNNT resin mixture in an orderly manner to obtain 87% filler composition. Afterward, the hardener, catalyst, and retardant were then merged with the obtained paste mixture and subjected to mechanical mixing once again to guarantee its uniform distribution. Subsequently, the mixture was then poured into a metal mold and cured in a hot-press machine under 120 °C for 2 h. To expound the contribution of the surface-treated BNNT in terms of thermal conductivity, samples without BNNT and neat BNNT incorporated were also prepared following the same method.

### 2.4. Characterizations

The morphological and organizational characteristics of the fabricated composites were examined through a field-emission scanning electron microscope (FESEM, CX-200 COXEM, Coxem (SK)), and the samples were prepared through a cross-sectional break in the composite upon exposure to liquid nitrogen. Fourier-transform infrared (FTIR, Perkin Elmer Spectrum Two FTIR-ATR, Thermo scientific (SK)) profiles were gathered with a 4/cm resolution and a heap of 32 scans in a 500–4000/cm range, and X-ray photoelectron spectroscopy (XPS, Axis-Supra, Kratos (MY)) using Mg Kα radiation was executed to foresee the functional groups on the surface-treated BNNT. The thermal permanency of the produced composites was scrutinized using a thermo-gravimetric analyzer (TGA), which was processed at a heating rate of 10 C/min in the 100–800 °C thermal range under a nitrogen atmosphere. The differential scanning calorimetry (DSC, Labsys Evo TG-DTA, Setaram (FR)) was used to confirm the glass transition temperature (Tg) of the fabricated samples. The isotropic thermal conductivity (ko, W/mK) was measured using a thermal analyzer (Hot-Disk thermal analyzer, TPS500S, Hot disk) to represent the thermal dissipation performance of the fabricated composites. A practical heat dissipation test was performed to demonstrate the heat transport capability of the prepared composites through a homemade heating device that replicates the heating as well as cooling of an actual electronic device in ambient conditions. The heat spread was then discerned by an infrared camera (FLIR-E390, (EST)). The surface charge change in the BNNT was investigated through zeta potential measurement (Nano ZSP-ZEN5602, Malvern (UK)). The mechanical strength of the prepared samples was acquired using a universal testing machine (UTM, Withlab Co., Ltd., With laboratory (SK)).

## 3. Results and Discussion

### 3.1. Chemical and Physical Characterization of BNNT-CAPA

**Figure 1:**
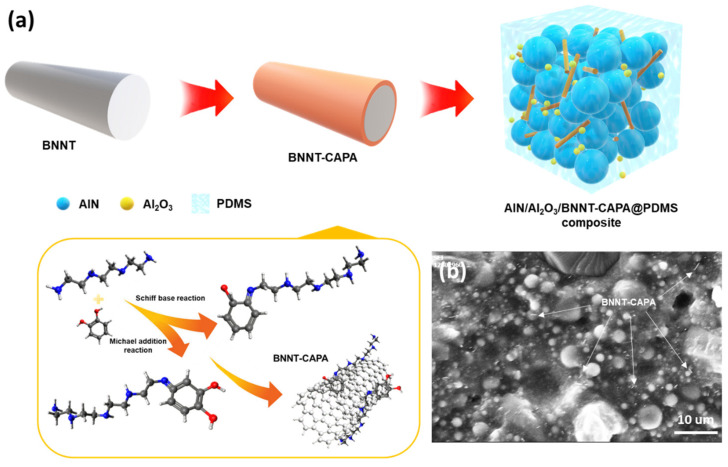
(**a**) Schematic representation of the preparation of BNNT-CAPA and (**b**) SEM image of PDMS/AlN/Al_2_O_3_/BNNT-CAPA composite.

**Figure 2:**
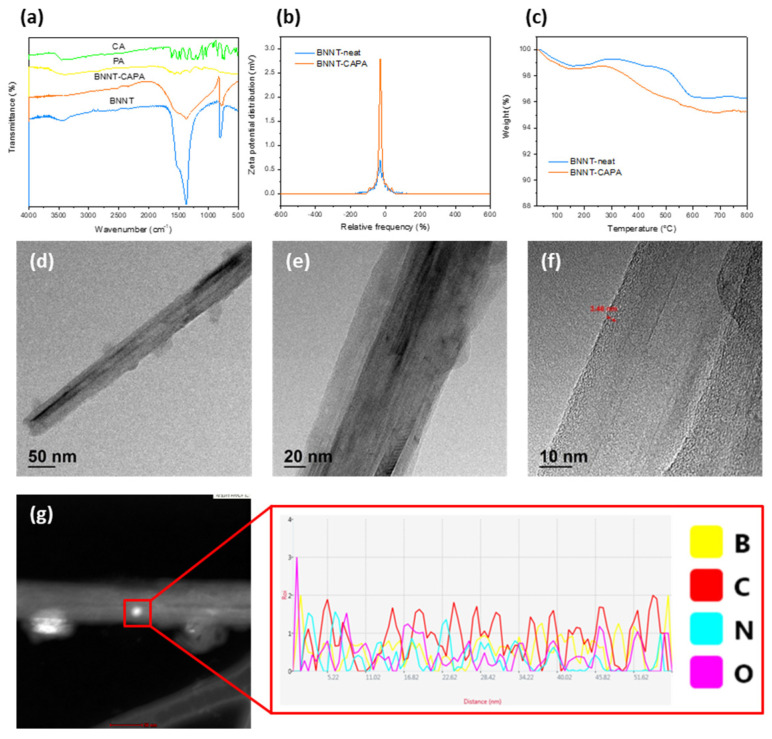
(**a**) FTIR spectra of CA, PA, BNNT-neat and BNNT-CAPA. (**b**,**c**) TGA and Zeta potential of BNNT-neat and BNNT-CAPA. (**d**–**f**) TEM image and (**g**) elemental line scan of BNNT-CAPA.

**Figure 3:**
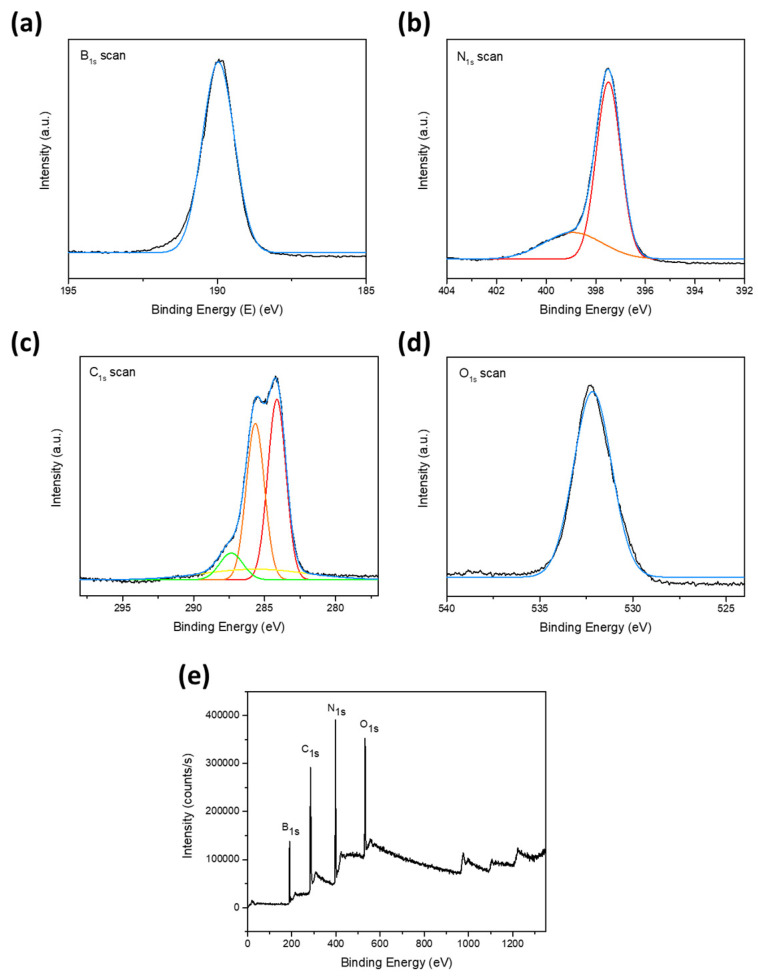
XPS survey of the surface-modified BNNT. (**a**) B 1s; (**b**) N 1s; (**c**) C 1s; (**d**) O 1s; (**e**) high resolution spectra.

Likewise, influenced by the deposition mechanism of the PDA monomer through oxidative self-polymerization and its ability to attach and form a homogeneous film coating on the interface of numerous materials, an amine-rich functionalization strategy on the surface through the deposition of catechol and polyamine on BNNT under an alkaline buffer solution to exhibit accentuated polymeric adhesion and compatibility was employed. Due to the similarity in the molecular structure of PDA towards CA and PA, its deposition mechanism will be of the same course, and the catechol will transform into a quinone structure through an oxidative reaction [20,22]. Upon the catechol transforming into a quinone structure through oxidative reaction, PA was assimilated through Michael’s addition reaction and Schiff base reaction, as detailed in Figure 1a [23,24]. The deposition of catechol moiety onto the BNNT surface was assisted by the polyphenol groups through covalent and noncovalent interaction, establishing an excellent adherence property. Illustrated in Figure 2a are the FTIR spectra of the functionalized BNNT and corresponding precursor materials. It is observed that the hydroxyl groups at 3500/cm on BNNT were weakened, which can be associated with the CAPA deposition. Likewise, an apparent broadening of 1400/cm and 800/cm peaks can be discerned after modification, asserting CAPA integration due to the manifestation of the hydrogen bonding and vibration frequency adjustment. Furthermore, XPS survey mapping was also performed to provide more insight into the elemental properties of the modified BNNT, as shown in Figure 3a–e. The B 1 s scan can be deconvoluted with binding energy 190.01 eV, which can be attributed to the B−N bond, which is in good accord with the standard XPS value of BNNT. The N 1 s spectrum indicates the existence of two peaks ascribed to the binding energies 397.20 eV, which can be ascribed to the N−B bond, and 399.89 eV, which corresponds to the N−C bond. The C 1 s scan indicates the presence of four distinct peaks with relatively different binding energies at 284.10, 285.60, 287.39, and 285.22, which can be ascribed to C−C, C−O, C−O, and C−N 20. The O 1 s spectrum was deconvoluted at 532 eV. The presence of the C−O bond signifies the manifestation of CAPA, thereby insinuating the successful interfacial modification of BNNT.

The structural attributes and elemental characteristics of BNNT before and after modification are presented in Figure 2d–g and Figure S1a–d through TEM images and TEM-EDX. It can be discerned that there is no apparent change in the morphological integrity of BNNT upon surface treatment, which showcases the aptitude of the proposed strategy for the interfacial modification of BNNT. An increase in the surface roughness on the functionalized BNNT can be distinguished, attesting to the successful deposition of CAPA, with a thickness of roughly ~3.4 nm. The presence of C and O denotes the successful deposition of the surface-treating agents onto the BNNT’s interface, which is analogous to the FTIR and XPS data. The domineering elemental profile of C affirms the presented functionalization strategy. Moreover, the surface characteristics of the BNNT before and after modification were also scrutinized to provide insight into the prospective behavior of BNNT, as presented in Figure 2b. A massive increase in surface charge can be realized from the surface charge measurement, which affirms the apparent deposition of CAPA onto the BNNT. The thermal stability attributes of the modified BNNT and neat BNNT were then assessed through TGA analysis, as shown in Figure 2c. Both samples follow the same degradation profile; hence, the neat BNNT showed a higher residue amount in comparison to BNNT-CAPA, and the difference in the residue amount can be ascribed to the CAPA coating on the modified BNNT, which is roughly ~1 wt %. The mechanical performance of the fabricated composite was also evaluated to observe the effect of BNNT-CAPA incorporation, as presented in Figure S2. The BNNT-CAPA assimilated samples demonstrated a maximum tensile strength of 4.4 Mpa, while the PDMS/AlN/Al_2_O_3_ obtained a tensile strength of 3.95 Mpa. A respective change in elongation was also observed with the PDMS/AlN/Al_2_O_3_/BNNT-CAPA. This change can be associated with the geometrical advantages of BNNT and its relative effective dispersion, and the surface treatment compatibility with the polymeric composite. This stipulates the relative contribution of BNNT on the thermal composite material [25].

### 3.2. Modified BNNT Dispersion and Thermal Conductivity Performance

**Figure 4:**
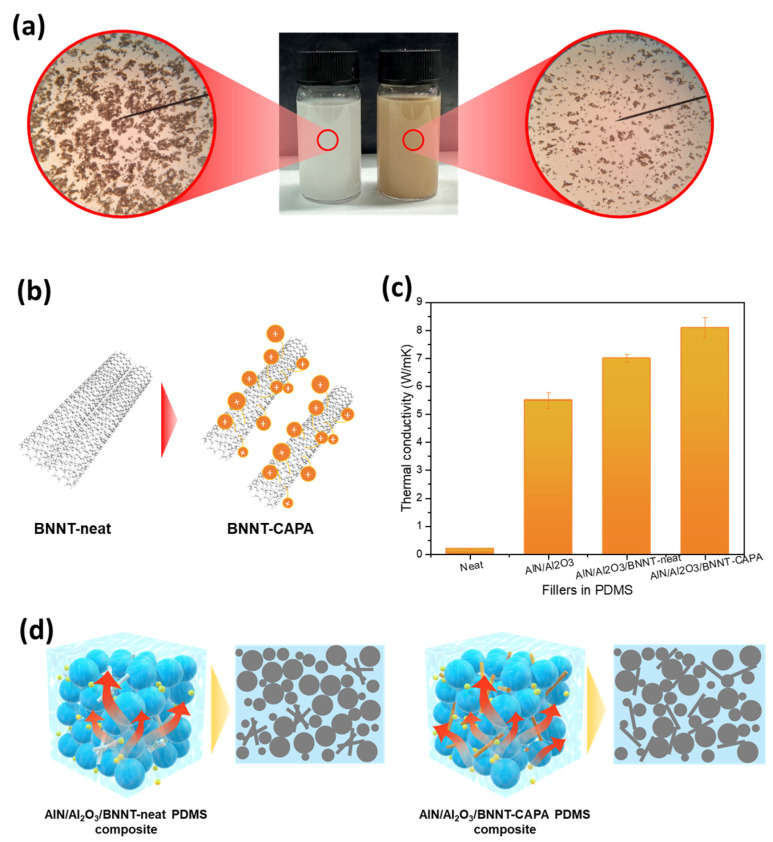
(**a**,**b**) Photograph and schematic representation of BNNT-neat and BNNT-CAPA dispersed in the solvent resin mixture and its corresponding (**c**) isotropic thermal conductivity of neat, AlN/Al_2_O_3_, AlN/Al_2_O_3_/BNNT-neat, and AlN/Al_2_O_3_/BNNT-CAPA. (**d**) Heat dissipation mechanism of the functionalized and non-functionalized BNNT incorporated composite and its corresponding phonon conduction pathways.

Presented in Figure 1a,b is a schematic representation of the surface modification of BNNT with catechol and polyamine and its corresponding composite assembly. The resulting composite is expected to demonstrate heightened thermal conductivity due to the introduction of BNNT by ensuring its effective construction of thermal pathways due to its dispersion improvement. It can be realized that the assimilation of 1 wt. %BNNT did not affect the structural arrangement of Al_2_O_3_ due to its micro/nanoscale attributes. To thoroughly scrutinize the dispersive contribution of BNNT’s surface modification, BNNT-neat and BNNT-CAPA were dispersed in a mixture of IPA solvent and PDMS resin, as shown in Figure 4a,b. An abundance of BNNT aggregates was observed on the sample with the neat BNNT, and this can be associated with the interfacial incompatibility of BNNT towards the polymeric matrix, attesting the Van der Wall interaction upon the nanotubes [26,27]. On the other hand, the catechol-polyamine functionalized BNNT displayed a better dispersive attribute, which asserts an enhancement in BNNT’s surface modification. The better dispersion of the modified BNNT rooted from the CAPA attachment onto the interface of BNNT leads to an electrostatic repulsive mechanism, discouraging nanotube clustering. This observation is in agreement with the zeta potential results, showing a higher surface charge on the BNNT-CAPA. The same can be observed upon the composite incorporation of BNNT-CAPA onto the polymeric matrix, as presented in Figure 1b, where the nanotubes can be sparingly discerned, which can be correlated to its augmented interface that is highly compatible with PDMS, easing its dispensability and abating the aggregation occurrence.

Shown in Figure 4c is the isotropic thermal conductivity of the prepared samples; the neat PDMS projects the lowest thermal conductivity of 0.19 W/mK, which is analogous to its standard value. A steep thermal conductivity increase was distinguished upon inorganic filler assimilation, obtaining a thermal conductivity value of 5.5 W/mK. A certain amount of (1% wt.) BNNT-neat and BNNT-CAPA was then introduced into the composite system, and both composites demonstrated an improved thermal conductivity, with BNNT-neat obtaining 7.0 W/mK, while BNNT-CAPA attained 8.1 W/mK. The difference between the results of the samples assimilated with BNNT is the respective dispersive properties of the modified BNNT relative to its neat counterpart, as illustrated in Figure 4d. It is postulated that the effective distribution of the nanofiller onto the composite matrix heightened the formation of phonon conduction networks that insinuate heat dissipative properties. Moreover, the deposited CAPA layer not only serves as a platform for the improvement in BNNT dispersion but also augments its interaction with the polymeric matrix, thereby enhancing the overall thermal conductivity. This also allows the modified BNNT to participate in the polymeric crosslinking reaction of the polymeric matrix due to the presence of the active groups on its interface. The surface modification also provides better interaction between the polymeric matrix and BNNT, instigating a reduction in interfacial resistance, thereby motivating seamless phonon movement [28]. In the course of the polymer crosslinking process, a strong interactive force may occur between the carboxyl groups and vinyl groups of PDMS and the amino groups and phenolic hydroxyl groups of BNNT-CAPA, thereby increasing the compatibility between the polymeric matrix and the BNNT. Moreover, to further highlight the contribution and advancement presented here, the relative comparative thermal dissipative performance is presented in Table S1. Most of these publications demonstrate roughly ~50–200% augmentation upon the incorporation of boron nitride. Hence, it should be noted that most of this research reports a high amount of boron derivatives to obtain enhanced thermal conductivity; therefore, the improvement contributions of boron nitride derivatives must be thoroughly discussed. The PDMS/AlN/Al_2_O_3_/BNNT-CAPA exhibited a 33.76% thermal conductivity enhancement per BNNT-CAPA volume percent incorporation. This can account for the effective dispersion of BNNT-CAPA, allowing the interparticle network formation to exploit the geometrical advantage of BNNT. Moreover, the presented strategy provides insights on the development of thermal materials utilizing boron nitride as an additive, addressing its economic limitations due to its high price.

Aside from the thermal conductivity, strong emphasis was directed towards the reduction in the overall thermal resistance for effective heat transfer. To analytically express the effect of BNNT-CAPA incorporation on the overall thermal resistance (R_O_), Equation (1) was used to generate heat flux transport efficiency across the bond line thickness (BLT) [29]. By setting the application area on all the samples constant, the following overall thermal resistance was observed, where the PDMS-neat polymer obtained the highest resistance, followed by the PDMS/AlN/Al_2_O_3_, PDMS/AlN/Al_2_O_3_/BNNT-neat, and PDMS/AlN/Al_2_O_3_/BNNT-CAPA (2.0 × 10^−3^ Km^2^/W, 1.0 × 10^−5^ Km^2^/W, 8.5 × 10^−6^ Km^2^/W and 7.4 × 10^−6^ Km^2^/W). These results affirm the network pathway contribution of BNNT-CAPA, serving as an interparticle connector for effective heat dissipation.

R_O_ = BLT/(A·k) (1)

**Figure 5:**
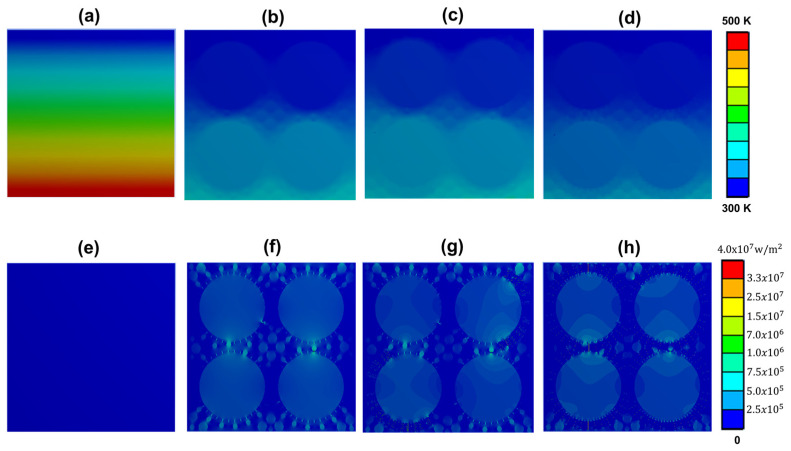
(**a**–**h**) Thermal evolution simulation during heat flux dissipation in the PDMS, PDMS/AlN/Al_2_O_3_, PDMS/AlN/Al_2_O_3_/BNNT and PDMS/AlN/Al_2_O_3_/BNNT-CAPA composite.

The heat transfer of the prepared composite was validated during heating to further enunciate the underlying mechanistic principle through ANSYS Fluent simulation. Presented in Figure 5a–h is the simulated thermodynamic propagation of PDMS-neat, PDMS/AlN/Al_2_O_3_, PDMS/AlN/Al_2_O_3_/BNNT-neat, and PDMS/AlN/Al_2_O_3_/BNNT-CAPA composites. The boundary condition of the simulation was set at 130 μm × 130 μm, with a direct heat source at the bottom transporting heat flux of 2 × 10^5^ W/m^2^ at 100 °C. This was set to mimic the thermal generation of electronic devices to correlate the thermal conductivity improvements toward the heat dissipation performance. The steady-state heat transfer equations (Equations (2) and (3)) were applied to calculate the temperature profile and heat flux inside the designed models.

∇∙q = 0(2)

q = −k∇T(3)

where q is the heat flux vector, T is the temperature, and k is thermal conductivity.

The thermal conductivity of the fillers was set at 0.17 W/mK, 30 W/mK, 120 W/mK, and 300 W/mK, corresponding to PDMS, AlN, Al_2_O_3_, and BNNT. The PDMS/AlN/Al_2_O_3_/BNNT-CAPA demonstrated the fastest heat dissipation on all the simulated composites, obtaining a 335 °C bottom interface temperature; this was followed by PDMS/AlN/Al_2_O_3_/BNNT-neat attaining 357 °C, and, lastly, with PDMS/AlN/Al_2_O_3_ (360 °C). These observations are analogous to the experimental results. Moreover, the PDMS/AlN/Al_2_O_3_/BNNT-CAPA composite exhibited homogeneous heating dissemination compared to its corresponding counterparts, which can be perceived from the heating gradient diffusion from the heat source towards the interface of the composite. This indicates the dominant heat transfer pathway construction of the BNNT-CAPA, which assists in the phonon transport and reduction in the interfacial thermal resistance [30].

### 3.3. Practical Thermal Management Demonstration Performance

As our technological dependence expands the continuous miniaturization and densification of electronic devices, an inevitable heat accumulation dilemma arises with it. Therefore, the development of high heat-dissipating thermal materials is imperative. Subsequently, to demonstrate the viability of the assembled composite for heat management applications, a heating device was developed to emulate the heat flux magnitude of chips on electronic devices, as shown in Figure S3

**Figure 6:**
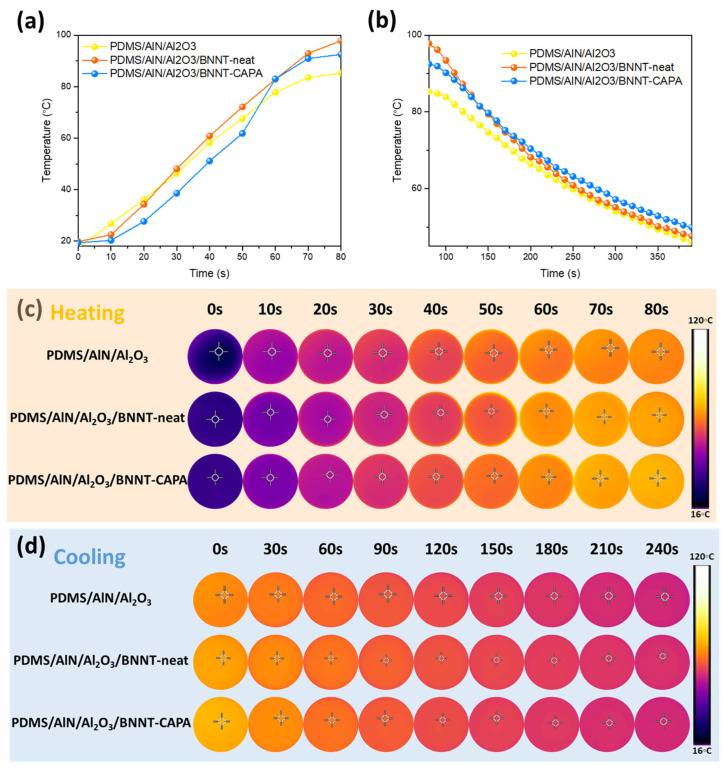
(**a**) Heating and (**b**) cooling profiles of neat and BNNT incorporated samples and (**c**,**d**) their corresponding thermal infrared images (the concentration of all the samples is 87% wt).

The corresponding heating and cooling profiles of the fabricated composite were examined and are shown in Figure 6a–d. All of the prepared samples demonstrated an increasing trend toward heat exposure, implying their viability to dissipate heat from the heat source. It can be recognized that the composite incorporated with BNNT-CAPA projects the highest heating profile, parallel to the thermal conductivity results, which can be associated with the effective continuous network establishment of the well-dispersed BNNT-CAPA. This validates the efficiency of the organized framework on the thermal flux dissipation. Due to the improved thermal conductivity of the BNNT-CAPA-incorporated composite, it exhibited the highest interfacial thermal elevation, reaching 98.4 °C after 80 s, while the BNNT-neat compounded and AlN/Al_2_O_3_-neat samples attained 93.7 °C and 79.8 °C. The relative increase in the interfacial temperature of the BNNT-CAPA assimilated samples indicates an improvement in the phonon pathway network formation due to the effective dispersion of BNNT. It should also be noted that the cooling propagation of the BNNT-CAPA samples demonstrates rapid cooling in comparison with the sample of that of BNNT-neat. This proves the superiority of the heat conduction pathway construction of the modified BNNT in comparison to its neat counterpart.

### 3.4. Fire Repellence Performance Demonstration of PDMS/AlN/Al_2_O_3_/BNNT-CAPA Composite

It was observed that these filler additives successfully improved the fire repellence of polymeric composites in comparison with incorporated organic/inorganic flame retardants [4]. Numerous efforts to enhance the thermal conductivity and fire retardancy of polymeric composites have been reported using different methodologies, such as the incorporation of flame-retardant-modified nanofillers [31]. Nevertheless, the coherent structurization and design of nanofiller improvements in thermal conductivity and flame resistance with a simplistic approach are still big challenges that need to be addressed [32]. A reasonable surface treatment approach is imperative to devise a polymeric composite with simultaneously good thermal conductivity and fire repellency.

**Figure 7:**
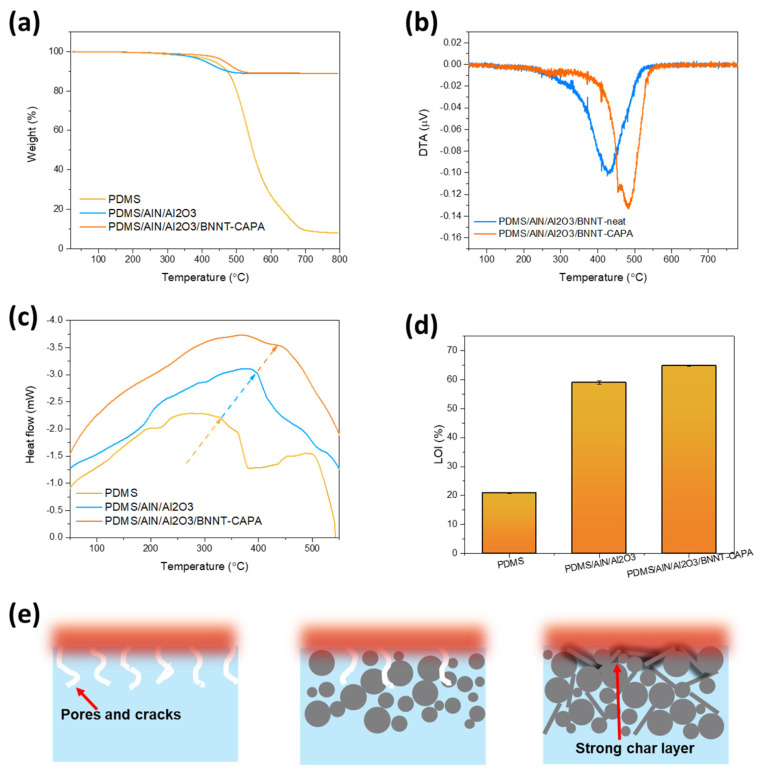
Flammability characteristic investigation using (**a**) LOI, (**b**) TGA, (**c**) differential thermal analysis (DTA) curves and (**d**) DSC and (**e**) the flame-retarding mechanism of BNNT-incorporated composite.

To evaluate the thermal stability and durability of the prepared composites, TGA and DTA assessments were employed, as displayed in Figure 7a,b. It is shown that the PDMS-neat underwent single-step degradation in the 500–700 °C temperature range, ascribed to the deterioration of PDMS networks, obtaining a char yield of 91.99%. It should be mentioned that the full degradation of the PDMS polymer can be associated with its transformation into a solid graphitic phase [33]. The incorporation of organic fillers massively decreases the char yield, obtaining 11.18% char residue. Moreover, the assimilation of BNNT-CAPA on the thermal stability of the composite improved, obtaining a char residue of 10.98%. The TGA results assert the durability contribution of BNNT-CAPA. Furthermore, DTA curves were formulated further to enunciate the thermal stability contribution of the modified BNNT. The heat resistance index (T_S_) was also calculated to explore the thermal stability characteristics of the cured resin using Equation (4). The temperature values were obtained at weight loss 5% (T_d5_) and 10% (T_d10_) of the samples from the TGA curve. Thermal degradation is important since most of the commercial thermal interface materials have 80–95% fillers. Both samples with and without BNNT-CAPA relatively lost ~12% of their corresponding weight. The BNNT-CAPA assimilated reached a T_S_ value of 242.44 °C, while the sample without BNNT-CAPA obtained 220.57 °C. The difference in the Ts can be ascribed to the ability of BNNT-CAPA to improve the physical heat tolerance of the composite material [34].

T_S_ = 0.49(T_d5_ + 0.6(T_d10_ − T_d5_) (4)

**Figure 8:**
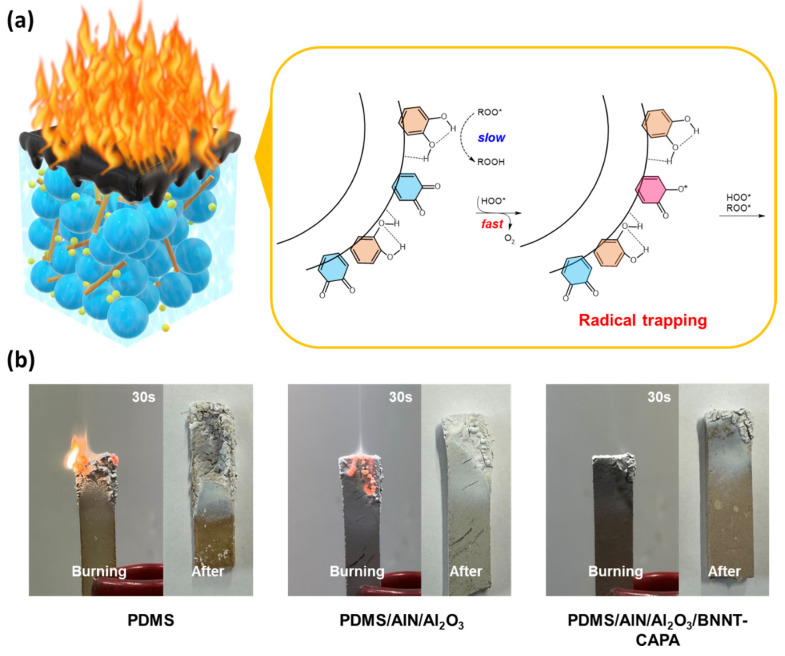
(**a**) Radical reactions between CAPA and regular free radicals (ROO* and HOO*). (**b**) Flame retardant of prepared composites.

It can be observed that a shift in the deterioration of temperature is observed upon the introduction of BNNT-CAPA, which affirms its thermal stability reinforcement contribution. This is also shown by the DSC measurements illustrated in Figure 7c, confirming the association between crystallinity alterations and the BNNT-CAPA incorporation. The phase change temperature of the prepared samples shows an increasing trend, relative to the following order: PDMS, PDMS/AlN/Al_2_O_3_, and PDMS/AlN/Al_2_O_3_/BNNT-CAPA. The glass transition propagation of pure PDMS samples was examined through DSC analysis and exhibited a Tm of 330.89 °C. Upon incorporation of the organic fillers, the Tm changed to 395.75 °C, with an enhancement of approximately 64.89 °C relative to PDMS-neat. The Tm was further increased by the assimilation of BNNT-CAPA, reaching up to 434.29 °C, with a relative elevation of 38.54 Tm in correlation with PDMS/AlN/Al_2_O_3_ samples. The melting point Tm of the BNNT-CAPA-integrated sample shows a massive increase relative to its small incorporating amount of 1%, showcasing its influence on the composite’s thermal durability properties, which can be ascribed to BNNT’s distinct thermal stability dominance and potent intrinsic permanence. The LOI trend is relatively synonymous with the TGA and DSC data, with LOI measurements of 20.8%, 58.97%, and 64.77%, corresponding to PDMS-neat, PDMS/AlN/Al_2_O_3_, and PDMS/AlN/Al_2_O_3_/BNNT-CAPA. The substantial augmentation in the thermal stability of BNNT-CAPA-incorporated samples is accredited to the following facets: effective dispersion, good charring capability, and radical trapping [35]. The effective dispersion of BNNT onto the polymeric matrix establishes a strong confinement effect due to its dominant nitrogen amount, protecting the mobility of polymeric networks [36]. A protective char is also heightened by the surface treatment, serving as a barrier that delays the penetration of oxygen and heat and inhibits the release of volatile components in the composite material, as shown in Figure 7e. Moreover, the attachment of the CAPA to the BNNT’s interface provides an additional supplementation, aside from its dispersive contribution. As a dopamine derivative, CAPA also possesses intrinsic fire repellence properties, imposing a radical scavenging effect, as demonstrated in Figure 8a [37,38]. The trapping of these free radicals imposed a suppression of the occurrence of further ignition and combustive reactions, thereby impeding the spread of fire [23]. This claim is analogous to the actual flame retardation shown in Figure 8b, where composite samples were exposed to fire and observed after 30 s. It can be recognized that the PDMS succumbed to flames, which asserts the LOI result, since its value is near the actual concentration of oxygen in atmospheric conditions. On the other hand, the sample infused with BNNT-CAPA established excellent fire repellency after flame exposure, in parallel to the discussed thermal stability analysis.

## 4. Conclusions

The surface modification of BNNT was effectively fused through a simplistic and straightforward process, achieving good dispersions. The improved BNNT dispersion forms an interconnected phonon pathway and the thermal interface resistance reduction due to its augmented compatibility with the polymeric matrix, thereby increasing the isotropic thermal conductivity of the prepared composite. Moreover, the articulated strategy exhibited a multi-functional effect for improving not only thermal conductivity but also fire retardancy. Incorporation of the BNNT-CAPA into the PDMS composite matrix increased the initial deterioration temperature and residual char yield, suggesting an improvement in the thermal stability. The incorporation of the BNNT-CAPA increased in LOI by about 10% relative to the pristine composite sample, revealing its contribution to the fire-inhibiting property of the composite. Analogously, the glass transition temperature obtained an increase of 38 C, which can be correlated with the synergy between the char formation from the combusted polymer and free radical scavenging effects. The apparent augmentation in the thermal conductivity and flame retardancy performance was essentially assigned to the overall dispersion improvement in BNNT-CAPA, highlighting the cooperative bilateral effect of BNNT and its surface modification onto the polymeric composite system. This work depicts a systematic strategy for the design and preparation of thermal interface material to achieve multifunctional materials for electric device applications.

## Data Availability

The data presented in this study can be requested from the corresponding author.

## Supplementary Materials

The following supporting information can be downloaded at: https://www.mdpi.com/article/10.3390/nano14100847/s1, Figure S1. TEM images of (a,b) pristine BNNT and (c,d) modified BNNT. (e) EDX elemental mapping profile. Figure S2. Stress and strain profile of the fabricated thermal composite. Figure S3. Experimental setup for the practical thermal management demonstration performance. Figure S4. Heat dissipation boundary conditions. (a) PDMS-neat, (b) PDMS/AlN/Al_2_O_3_, (c) PDMS/AlN/Al_2_O_3_/BNNT-neat and (c) PDMS/AlN/Al_2_O_3_/BNNT-CAPA. Table S1. Thermal conductivity performance of boron nitride-based composite and its percent improvement contribution. References [39,40,41,42,43,44] are cited in the Supplementary Materials.
