# Supplementary material for: Preparations and Thermal Properties of PDMS-AlN-Al2O3 Composites through the Incorporation of Poly(Catechol-Amine)-Modified Boron Nitride Nanotubes
Source: Nanomaterials (Basel). 2024 May 13;14(10):847. doi: 10.3390/nano14100847 (PMC11123707; doi:10.3390/nano14100847)
Supplement: Supplementary file 1 [file nanomaterials-14-00847-s001.zip › nanomaterials-2971796-supplementary.pdf]

Preparations and Thermal Properties of PDMS-AlN-Al_2O_3 Composites Through the Incorporation of Poly(Catechol-Amine) Modified Boron Nitride Nanotubes

Arni Gesselle M. Pornea[1], Dinh Duy Khoe[1], Zahid Hanif[1], Numan Yanar[1], Ki-In Choi[1], Min Seok Kwak[2], and Jaewoo Kim[1]*

[1] R&D Center, Naieel Technology, 6-2 Yuseongdaero 1205, 2nd FL, Daejeon 34104, Republic of Korea

[2] CMT Co., Ltd., 322 Teheran-ro, Hanshin Intervalley 24 Esat Bldg., Gangnam-gu, Seoul 06211, Republic of Korea

***** Correspondence: kimj@naieel.com

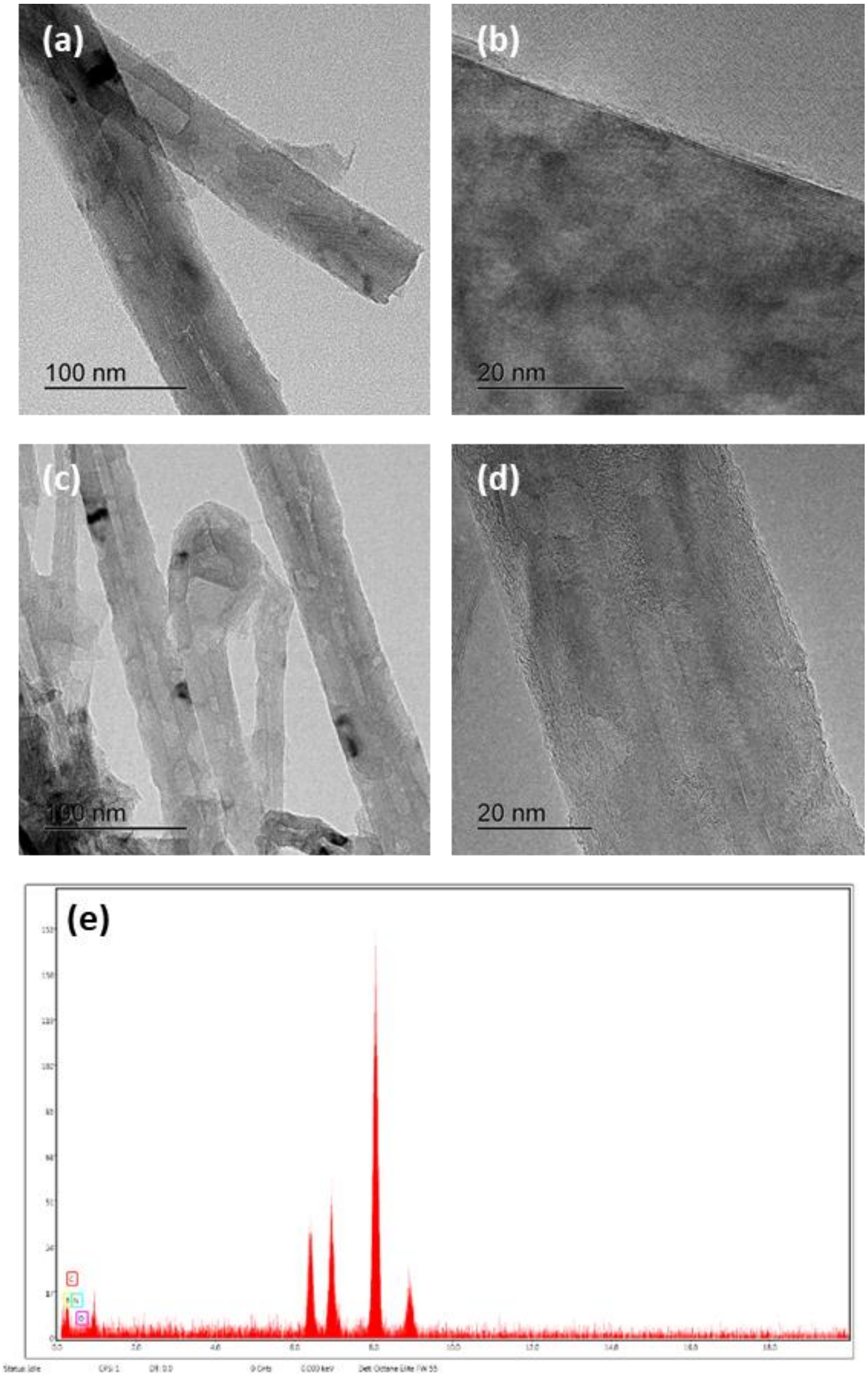

Figure S1. TEM images of (a-b) pristine BNNT and (c-d) modified BNNT. (e) EDX elemental mapping profile.

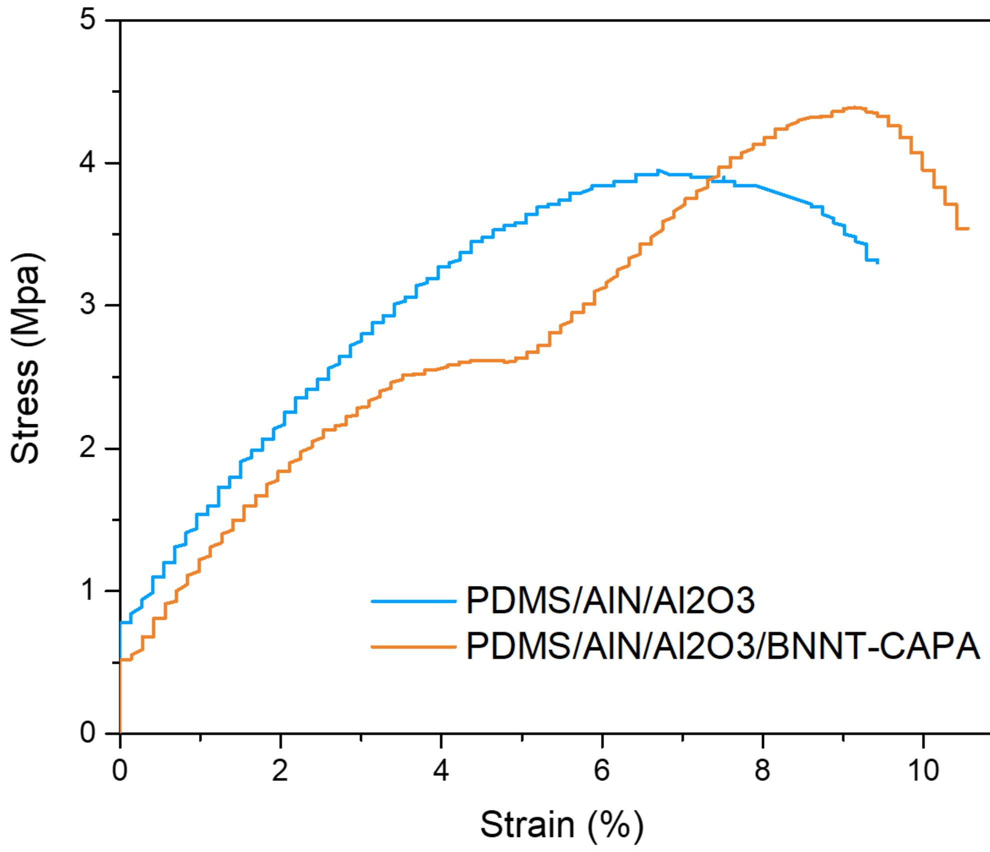

Figure S2. Stress and strain profile of the fabricated thermal composite.

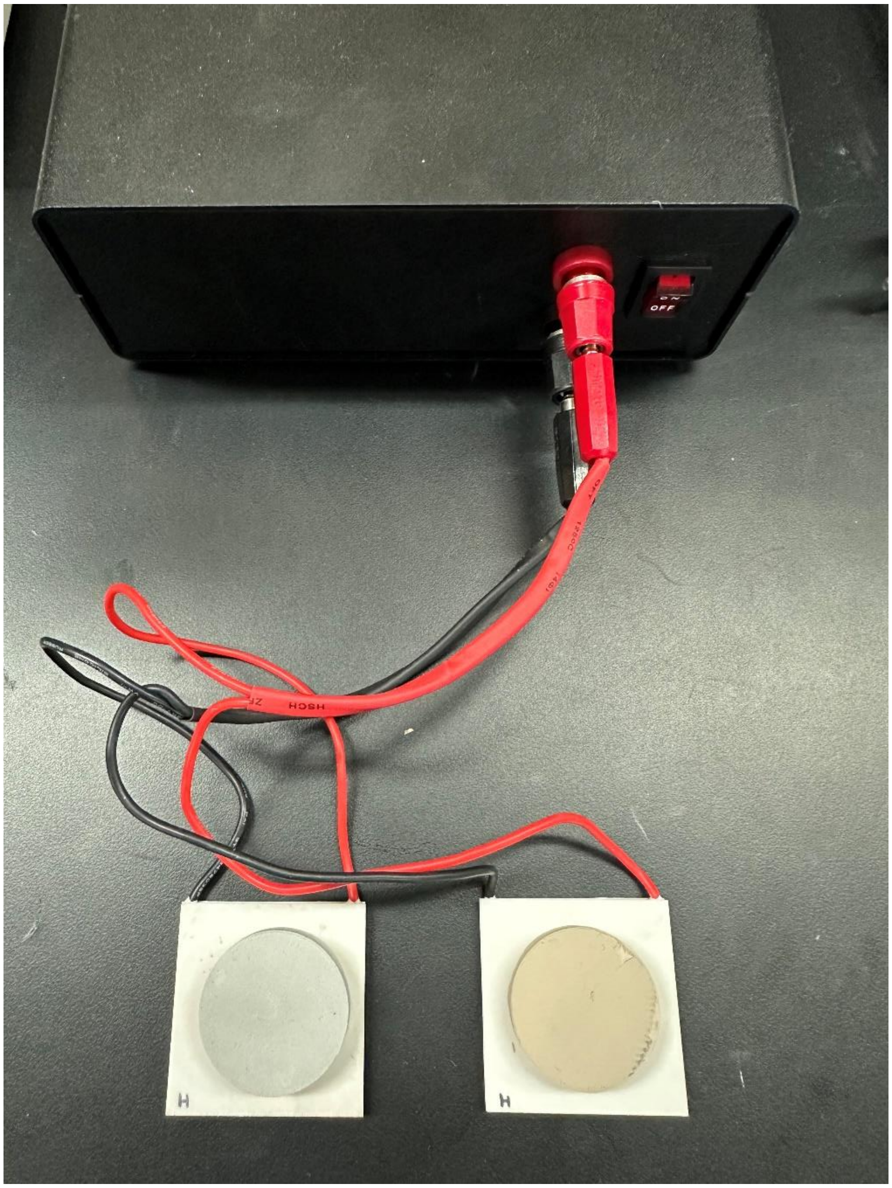

Figure S3. Experimental setup for the practical thermal management demonstration performance.

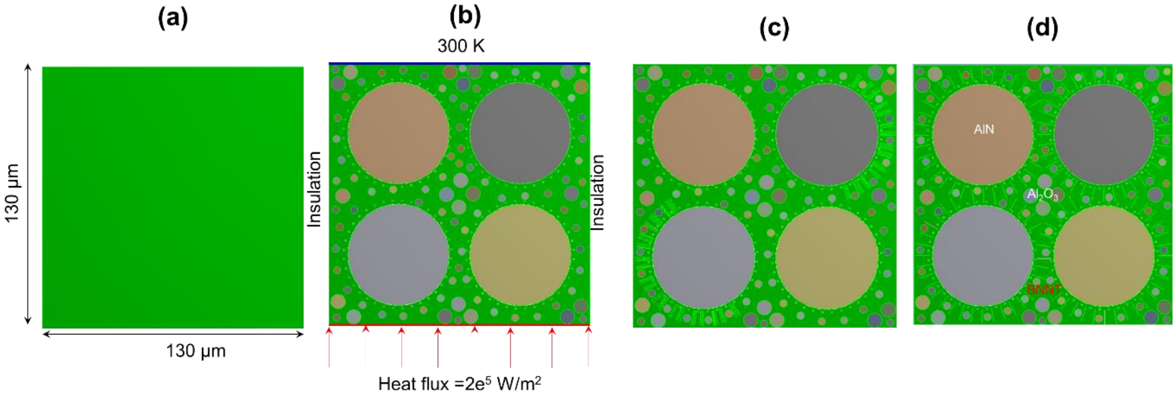

Figure S4. Heat dissipation boundary conditions. (a) PDMS-neat, (b) PDMS/AlN/Al_2O_3, (c) PDMS/AlN/Al_2O_3/BNNT-neat and (c) PDMS/AlN/Al_2O_3/BNNT-CAPA.

Table S1. Thermal conductivity performance of boron nitride-based composite and its percent improvement contribution

Polymer	Main filler	Boron nitride derivative	Thermal conductivity (W/mK)	Improvement effect per volume of boron nitride added (%)	Refs
Epoxy	Al_2O_3	hBN	4.40	8.80%	[1]
PDMS	Al_2O_3	hBN	3.50	11.11%	[2]
PI	Al_2O_3	hBN	3.35	4.38%	[3]
Epoxy	AlN	hBN	2.40	1.78%	[4]
PMMA	SiO_2	hBN	5.58	10.00%	[5]
Epoxy	Cubic boron nitride	hBN	5.20	15.96%	[6]
PDMS	AlN, Al_2O_3	BNNT	8.10	33.76%	This work

References

1. Mai, V.D.; Lee, D. Il; Park, J.H.; Lee, D.S. Rheological Properties and Thermal Conductivity of Epoxy Resins Filled with a Mixture of Alumina and Boron Nitride. *Polymers (Basel)* **2019**, *11*, doi:10.3390/polym11040597.

2. Kim, Y.K.; Chung, J.Y.; Lee, J.G.; Baek, Y.K.; Shin, P.W. Synergistic Effect of Spherical Al2O3 Particles and BN Nanoplates on the Thermal Transport Properties of Polymer Composites. *Compos Part A Appl Sci Manuf* **2017**, *98*, 184–191, doi:10.1016/j.compositesa.2017.03.030.

3. Liu, D.; Ma, C.; Chi, H.; Li, S.; Zhang, P.; Dai, P. Enhancing Thermal Conductivity of Polyimide Composite Film by Electrostatic Self-Assembly and Two-Step Synergism of Al2O3microspheres and BN Nanosheets. *RSC Adv* **2020**, *10*, 42584–42595, doi:10.1039/d0ra08048a.

4. Liang, D.; Ren, P.; Ren, F.; Jin, Y.; Wang, J.; Feng, C.; Duan, Q. Synergetic Enhancement of Thermal Conductivity by Constructing BN and AlN Hybrid Network in Epoxy Matrix. *Journal of Polymer Research* **2020**, *27*, doi:10.1007/s10965-020-02193-3.

5. Tang, Y.; Xiao, C.; Ding, J.; Hu, K.; Zheng, K.; Tian, X. Synergetic Enhancement of Thermal Conductivity in the Silica-Coated Boron Nitride (SiO2@BN)/Polymethyl Methacrylate (PMMA) Composites. *Colloid Polym Sci* **2020**, *298*, 385–393, doi:10.1007/s00396-020-04617-4.

6. Zhang, Y.; Gao, W.; Li, Y.; Zhao, D.; Yin, H. Hybrid Fillers of Hexagonal and Cubic Boron Nitride in Epoxy Composites for Thermal Management Applications. *RSC Adv* **2019**, *9*, 7388–7399, doi:10.1039/c9ra00282k.
